# Improvement of the Ultrasound-Assisted Extraction of Polyphenols from Welsh Onion (*Allium fistulosum*) Leaves Using Response Surface Methodology

**DOI:** 10.3390/foods11162425

**Published:** 2022-08-12

**Authors:** Carolina Medina-Jaramillo, Edward Gomez-Delgado, Alex López-Córdoba

**Affiliations:** Grupo de Investigación en Bioeconomía y Sostenibilidad Agroalimentaria, Escuela de Administración de Empresas Agropecuarias, Facultad Seccional Duitama, Universidad Pedagógica y Tecnológica de Colombia, Carrera 18 con Calle 22, Duitama 150461, Colombia

**Keywords:** *Allium fistulosum* L., antioxidant activity, ultrasound-assisted extraction, response surface methodology, optimization, polyphenols

## Abstract

Welsh onion (*Allium fistulosum*) leaves contain several bioactive compounds that can be extracted and used to develop new value-added products (e.g., functional foods and dietary supplements). In the current work, optimal ultrasound-assisted extraction (UAE) conditions to obtain extracts with high polyphenols content and DPPH (1,1-diphenyl-2-picrylhydrazil) scavenging activity were identified using response surface methodology. A complete 3^k^ factorial design was used to evaluate the effect of different variables of the UAE (extraction temperature, time, and ethanol concentration) on the polyphenols content and the DPPH scavenging activity of the extracts. The best conditions for UAE to reach both the highest values of total polyphenols content (51.78 mg GAE/100 g) and DPPH scavenging activity (34.07 mg Trolox equivalents/100 g) were an extraction temperature of 60 °C, time of 10 min, and ethanol concentration of 70% *v/v*. The antioxidant activity of the extracts obtained at the optimal conditions was also evaluated by 2,2′-azino-bis-3-ethylbenzthiazoline-6-sulphonic acid (ABTS) and ferric reducing antioxidant power (FRAP) assays obtaining values of 155.51 ± 2.80 μM Trolox/100 g and 1300.21 ± 65.55 μM Trolox/100 g, respectively. Moreover, these extracts were characterized by UHPLC-ESI+-Orbitrap-MS analysis finding that cyanidin (6.0 mg/kg) was the phenolic compound found in the highest amount followed by quercetin-3-glucoside (4.4 mg/kg).

## 1. Introduction

The cultivation and production of *Allium* species are commercial and economic activities which are of great importance for the agro-food industry [1]. They are one of the most produced and consumed vegetables worldwide. The world production of *Allium* species in 2020 was 32.4 × 10^6^ metric tons, with China being the country with the highest global contribution (~85% of world production) [1].

The *Allium* genus includes several species being the best-known *Allium cepa* (onion bulb), *Allium fistulosum* (Welsh onion), *Allium sativum* (garlic), *Allium porrum* (leek), and *Allium tuberosum* (Chinese onion) [2]. Among them, the Welsh onion is one of the most widely consumed worldwide because of its aroma, flavor, and medicinal properties [3,4]. This vegetable is mainly marketed in fresh or processed as sauces, paste, or dehydrated powders [5,6]. Additionally, in past years, the global market for minimally processed Welsh onions has been growing quickly [7].

The plant of Welsh onion is comprised of three fundamental parts, i.e., shoots, leaves, and non-developed bulbs [5]. In particular, the leaves represent around 23% of its fresh weight. Several studies have reported that the Welsh onion leaves are a source of carbohydrates (6.5 g/100 g), protein (1.9 g/100 g), minerals (Ca, Mg, P, Fe, and Zn), and vitamins (vitamin A, thiamine, ascorbic acid, and folic acid) [5]. Moreover, they are rich in bioactive compounds including phenolic acids and flavonoids [3,8,9]. Some of these compounds have been associated with their antioxidant, antimicrobial, anti-inflammatory, and hypo-cholesterolemic properties [10,11]. Furthermore, the cytotoxic activity of *Allium fistulosum* extracts and their isolated phenolic compounds has been also evaluated, finding that they are effective against cancer cell lines such as MCF-7 and HepG 2 [12,13,14].

There are some studies about the extraction of beneficial compounds from Welsh onions leaves, mainly using the conventional extraction technique which is based on the maceration of the leaves using different solvents (e.g., ethanol, ethanol/water, acetone), times, and temperatures [3,15,16,17]. This method takes a lot of time, energy, and solvent during processing. Therefore, the interest of researchers in new non-conventional extraction methods such as UAE has been growing in recent years [18]. UAE is based on the utilization of ultrasonic energy (sound waves with frequencies more than 20 kHz) to facilitate the extraction of analytes from a solid sample by the solvent [19,20]. The use of UAE offers advantages over the conventional method such as selectivity, low energy consumption, reduction in solvent consumption, and extraction time, and reduction in consumption of hazardous chemicals, among others [15,21,22,23]. UAE has been used to extract phenolic compounds from different matrices, including *Achillea arabica* [24], spruce wood bark [25], *Jatropha dioica*, *Eucalyptus camaldulensis* [26], black chokeberry [27], and others [28,29,30,31]. However, few studies have been conducted on the UAE of polyphenolic compounds from Welsh onion leaves.

This research aimed to identify optimal ultrasound-assisted extraction (UAE) conditions (ethanol concentration, time, and temperature) for the obtention of extracts from Welsh onion leaves, with high polyphenols content and DPPH scavenging activity, using response surface methodology.

## 2. Materials and Methods

### 2.1. Materials

All solvents and reagents were of analytical grade. Ethanol (70% *v/v*) and sodium carbonate (Na_2_CO_3_) were supplied by Loba chemie (Mumbai, India). Folin-Ciocalteu reagent and the DPPH free radical were provided by Panreac (Barcelona, Spain). Gallic acid was provided by Merck (Darmstadt, Germany). 6-Hydroxy-2,5,7,8-tetramethylchroman-2-carboxylic Acid (Trolox), ABTS and TPTZ (2,4,6-tripiridil-striazina) were provided by Sigma Aldrich (St. Louis, MO, USA).

UHPLC-grade standards were purchased from Sigma Aldrich: caffeine, theobromine, theophylline, (±)-catequina, (−)-epigallocatechin gallate, (−)-epicatechin, (−)-epicatechin gallate, (−)-epigallocatechin, caffeic acid, p-coumaric acid, ferulic acid, rosmarinic acid, quercetin, naringenin, luteolin, kaempferol, pinocembrin, apigenin, cyanidin 3-rutinoside, pelargonidin 3-glucoside, quercetin-3-glucoside.

### 2.2. Sample Preparation

Fresh Welsh onions (*Allium fistulosum* L.) were purchased at a local market in the city of Duitama (Boyacá, Colombia). Prior to use, the vegetables were washed with a 100 mg L^−1^ NaClO solution, peeled, trimmed (roots and leaf tips cut), and rinsed with water [7]. The trimmed leaves were cut into pieces of 1 cm, frozen at –20 °C for 24 h, and dried using a BUCHI Lyovapor L-200 freeze dryer (Flawil, Switzerland) operating at −55 °C and a chamber pressure of 0.1 mbar for 48 h. The freeze-dried cakes were chopped using an electrical grounder and sieved through a 30-mesh sieve (710-μm aperture size) to obtain fine powders.

### 2.3. Ultrasound-Assisted Extraction

Ultrasound-assisted extraction was carried out in a BRANSON CPX 1800 ultrasonic bath (Bransonic, Danbury, CT, USA). This consisted of a rectangular ultrasound chamber (length:10 cm, height:12 cm, depth: 12 cm) with industrial transducers of 40 kHz and a power of 70 W [32]. The ultrasound chamber was kept at a constant temperature by circulating water from a thermostatic bath (Memmert WNB14, Schwabach, Germany).

Samples (2.4 g) of ground Welsh onion leaves were placed in capped tubes (20 mL) and blended with 3 mL of extraction solvent according to the experimental design (Table 1). Then, the tubes with the blends were immersed in the ultrasound chamber at a fixed position and sonicated in a continuous mode for different times (Table 1). All the experiments were performed at a power density of 23.3 W/mL. The resulting extracts were filtered and kept refrigerated (4–6 °C) until use.

### 2.4. Total Polyphenols Content

The Folin–Ciocalteu assay was used to determine the total polyphenols content of the extracts [33]. Briefly, 400 μL of each extract were blended with 2 mL of Folin–Ciocalteu reagent (1:10 diluted). Then, 1.6 mL of Na_2_CO_3_ (7% *w/v*) were added to each sample. After 30 min, the absorbance was measured at 760 nm using a spectrophotometer (X-ma1200 Human Corporation, Loughborough, UK). Calibration curves were prepared using aqueous solutions of gallic acid in the concentration range of 4–90 mg/L. The method validation was carried out according to ICH Guideline (Supplementary Material Table S1) [34]. The results were expressed as mg of gallic acid equivalents (GAE) per 100 g of sample (mg GAE/100 g).

### 2.5. Antioxidant Activity

The antioxidant activity of the extracts from Welsh onion leaves was evaluated by the DPPH, FRAP and the ABTS assays.

#### 2.5.1. DPPH Assay

DPPH assay was carried out according to the method described by Brand-Williams [35]. A volume of 100 μL of each sample was mixed with 3.9 mL of DPPH ethanol solution (25 mg DPPH/L). Absorbance was determined at 515 nm until the reaction reached a plateau. The calibration curve was carried out with Trolox standard in the concentration range of 10–170 mg/L. The validation parameters are shown in Supplementary Material Table S1. The results were expressed as mg Trolox equivalents (Trolox) per 100 g of sample (mg Trolox/100 g).

#### 2.5.2. ABTS Assay

ABTS test was carried out in accordance with the procedure described by Re et al. [36]. ABTS radical cation (ABTS^•+^) was produced by reacting 7 mM ABTS stock solution with 2.45 mM potassium persulfate and allowing the mixture to stand in the dark at room temperature for 16 h before use. Then, the ABTS^•+^ solution was diluted with ethanol until it had an absorbance of 0.70 at 734 nm. Samples of 10 µL of ethanolic extract were mixed with 1.0 mL of diluted ABTS^•+^ solution and the absorbance was measured after 6 min of reaction. The calibration curve was carried out with Trolox standard in the concentration range of 10–2000 μM. The validation parameters are shown in Supplementary Material Table S1. The results were expressed as μM Trolox per 100 g of sample (μM Trolox/100 g).

#### 2.5.3. FRAP Assay

FRAP assay was conducted following the method described by Benzie et al. [37]. The FRAP reagent was prepared by mixing sodium acetate buffer solution (300 mM), TPTZ (10 mM in 40 mM of HCl), and ferric chloride hexahydrate (20 mM) at a ratio of 10:1:1, respectively. Freshly prepared FRAP reagent (2.9 mL) was mixed with 100 µL of each sample. The resulting mixtures were incubated for 30 min at 37 °C and absorbance was measured at 593 nm. The calibration curve was prepared with Trolox standard in the concentration range of 100–500 μM. The validation parameters are shown in Supplementary Material Table S1. The results were expressed as μM Trolox per 100 g of sample (μM Trolox/100 g).

### 2.6. UHPLC-ESI+-Orbitrap-MS Analysis

UHPLC-ESI+-Orbitrap-MS analysis was performed using the optimized protocol developed by Stashenko et al. [38]. The extracts from Welsh onion leaves obtained by UAE were analyzed using an UHPLC Dionex™ UltiMate™ 3000 (Thermo Fisher Scientific, Sunnyvale, CA, USA), equipped with a binary pump (HP G3400RS), an automatic sample injector (WPS 300TRS) and a thermostatic unit for a TCC 3000 column. The interface of LC-MS was electrospray ionization (ESI). The spectrometer Orbitrap had high mass resolution with ion current detecting system. Chromatography separation was conducted by Hypersil GOLD aQ column of 100 × 2.1 mm, 1.9 μm of particle size (Thermo Scientific, Sunnyvale, CA, USA) at 30 °C.

The mobile phase was as follows: mobile phase A: 0.2% aqueous solution of ammonium formate; mobile phase B: 0.2% on acetonitrile ammonia formate. The initial gradient condition was 100% A, progressed linearly towards 100% of B in 8 min. Such a phenomena was held for 4 min, then returned to the initial conditions in one minute. The total running time lasted 13 min, plus 3 post-running additional minutes.

The compound identification was achieved by using the full scan mode and ion extraction (EIC) corresponding to the [M+H]^+^ of the target compounds. A mass assessment was performed with the accuracy and precision of ∆ppm < 1 and using a standard mix solution of phenolic compounds. The calibration curves were prepared using the UHPLC-grade standards above mentioned.

### 2.7. Experimental Design

The optimal combination of extraction variables was determined using a complete factorial design (3^3^) with three factors and three levels (Table 1). The factors studied were ethanol concentration (0, 35 and 70 % *v/v*), time extraction (10, 20 and 30 min), and temperature (30, 45 and 60 °C). These conditions were chosen based on preliminary experiments and previous works [26]. The selection of ethanol as the extraction solvent was made since it is inexpensive, non-toxic, and has uses in the food industry [39].

The response surface methodology (RSM) was performed using the Statgraphics XVI version 16.0 statistical software (Warrenton, VA, USA) to optimize the extraction of phenolic compounds and the DPPH scavenging activity. The optimal UAE conditions were determined by the regression analysis conducted on the data for the dependent variable obtained and were fitted to the following empiric polynomial model:

(1) $$Y_{i}=A_{o}+A_{1}x_{1}+A_{2}x_{2}+A_{3}x_{3}+A_{4}x_{1}x_{2}+A_{5}x_{1}x_{3}+A_{6}x_{2}x_{3}+A_{7}x_{1}{}^{2}+A_{8}x_{2}{}^{2}+A_{9}x_{3}{}^{2}$$

where *Y*_i_ is the response variable (total phenolics compounds (TPC) or DPPH scavenging activity); *A*_0_ is the intercept; *A*_1_, *A*_2_, and *A*_3_ are the linear regression coefficients of ethanol concentration (*x*_1_), extraction time (*x*_2_), and temperature (*x*_3_), respectively; *A*_4_, *A*_5_ and *A*_6_ are interaction regression coefficients; *A*_7_, *A*_8_ and *A*_9_ are quadratic regression coefficients.

An analysis of variance (ANOVA) was carried out at a significant level of 95% to evaluate the statistical significance of the parameters of the model.

### 2.8. Scanning Electron Microscopy (SEM)

The morphological characteristics of the onion leaves before and after the UAE processing were examined using a ZEISS EVO MA10 microscope (Carl Zeiss SMT Ltd., Cambridge, UK). The samples were fixed on stubs, covered with a layer of gold, and examined using an acceleration voltage of 20 kV.

### 2.9. Fourier Transform Infrared Spectroscopy (FTIR)

FTIR analysis was conducted in a Perkin-Elmer Spectrum TwoTM IR spectrometer (Waltham, MA, USA) equipped with attenuated total reflectance (ATR) module. The samples were mounted on the ATR accessory and analyzed under transmission mode, taking 64 scans per experiment with a resolution of 4 cm^−1^, over the region from 4000 to 600 cm^−1^.

### 2.10. Statistical Analysis

Statgraphics XVI version 16.0 software (Warrenton, VA, USA) was used for the statistical analysis. An analysis of variance and Tukey’s pairwise comparisons were performed with a 95% confidence level. The studies were performed at least in triplicate, and the results were given as mean ± standard deviation.

## 3. Results and Discussion

**Figure 1:**
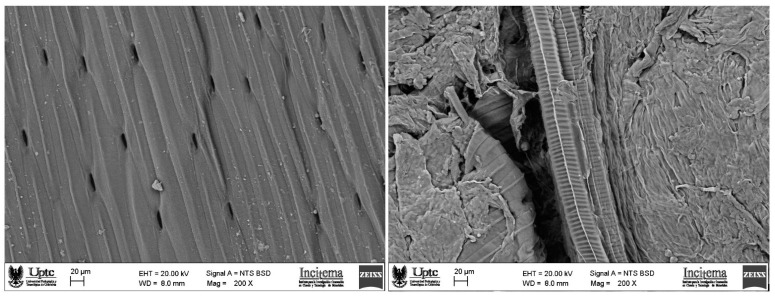
SEM images of Welsh onion leaves before (**left**) and after (**right**) ultrasonic processing.

Figure 1 shows SEM micrographs of the Welsh onion leaves before and after the ultrasound-assisted extraction (UAE). It was observed that the ultrasonic processing caused structural changes in the Welsh onion leaves provoking cell wall fragmentation and the microcracks formation. This behavior could be due to the acoustic cavitation that has been reported as the main mechanism involved in the UAE [19,20,40]. Similar results were reported by Altemini et al. when they studied the structural changes for pumpkin and peach samples before and after UAE [31].

Table 1 shows the total polyphenols content (TPC) and the DPPH scavenging activity of the extracts from Welsh onion leaves obtained from all of the experiments. The extracts showed a polyphenols content ranging from 16.87 to 53.83 mg GAE/100 g and a DPPH scavenging activity ranging from 13.09 to 33.78 mg Trolox/100 g. Values of total polyphenols content of ~15.82 mg/100 g and DPPH scavenging activity of ~37.88 mg Trolox/100 g have been reported for *Allium fistulosum* [8] and *Allium roseum* [41], respectively.

### 3.1. Effect of the UAE Conditions on Total Polyphenols Content

**Figure 2:**
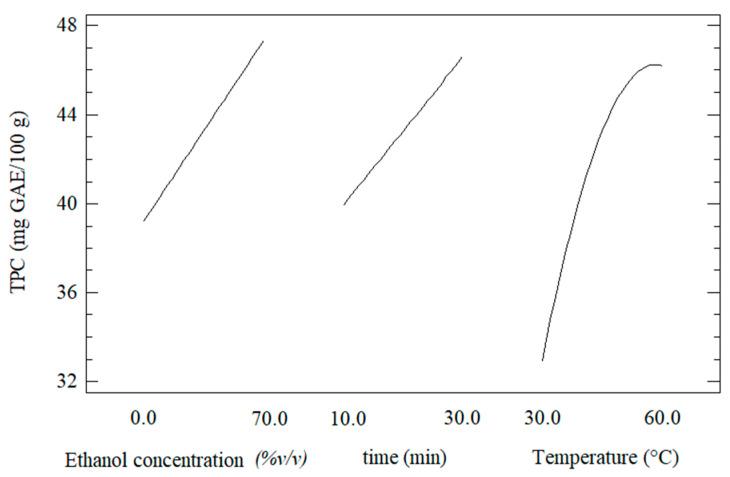
Effect of the individual variables process on the total polyphenols content (TPC) of the extracts from Welsh onion leaves.

Figure 2 shows the individual effect of the UAE conditions on the total polyphenols content of the extracts from Welsh onion leaves. It was observed that the extraction of phenolic compounds was increased at higher values of ethanol concentration, time extraction, and temperature. This behavior was attributed to that an increase in the ethanol concentration allows more polyphenols to dissolve and diffuse through the solid matrix of Welsh onion leaves. This mass transfer could increase if the residence time for diffusion is more significant [42]. Moreover, it has been reported that an increase in the temperature extraction commonly favors the solubility of polyphenols in ethanol and decreases the viscosity of the ethanol-polyphenols solution, favoring diffusion through the cellular matrix, making liquid-solid extraction more effective [30,42].

Equation (2) shows the fitted second-order polynomial model for the total polyphenols content (TPC) obtained from the response surface methodology.

(2) $$TPC=-49.7151+0.1155x_{1}+1.7835x_{2}+2.5721x_{3}-0.0323x_{2}x_{3}-0.0165x_{3}{}^{2}$$

The results showed that the fitted model for total polyphenols content had adjusted R^2^ around 72.82%. On the other hand, ANOVA indicated that the three main factors (ethanol concentration (*x*_1_), extraction time (*x*_2_), and temperature (*x*_3_)) and the interactions between time-temperature (*x*_2_ *x*_3_) and temperature-temperature (*x*_3_^2^) were the most significant parameters for obtaining an extract with a higher TPC content (*p* < 0.05) (Table 2). In contrast, the terms *x*_1_*x*_2_, *x*_1_*x*_3_, *x*_1_^2^, and *x*_2_^2^ were not significant and therefore they were eliminated for the model fitting (Equation (2)).

**Figure 3:**
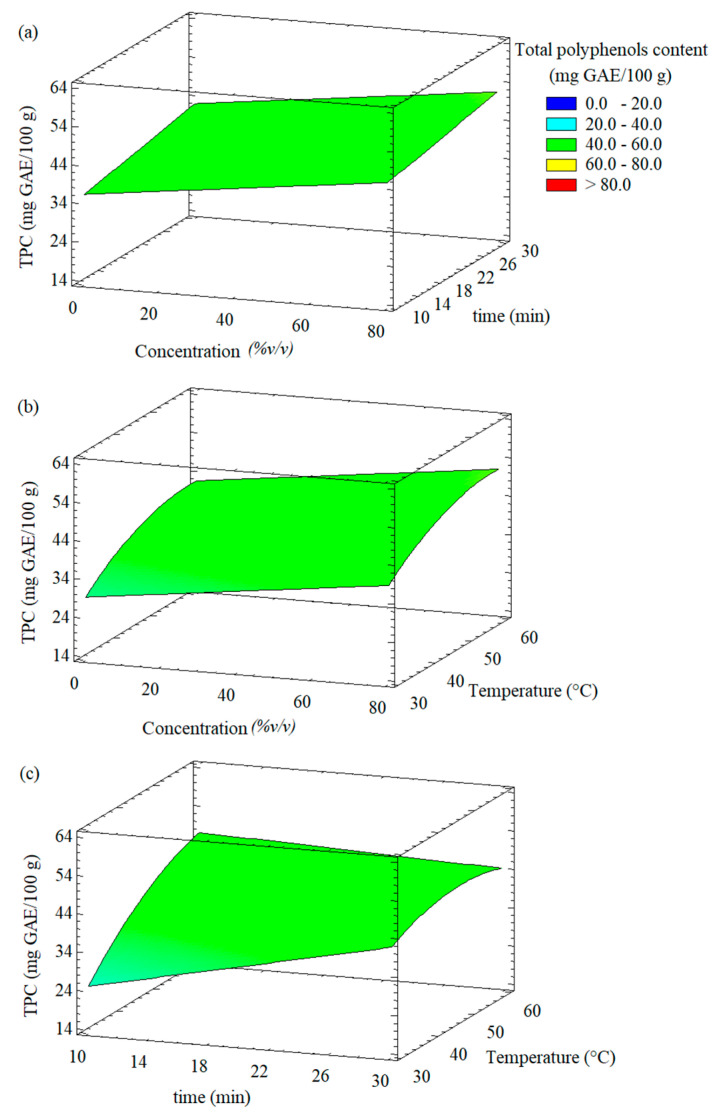
Response surface plots showing the effect of different experimental factors on the total polyphenols content (TPC) of the extracts from Welsh onion leaves: (**a**) effect of ethanol concentration and time; (**b**) effect of ethanol concentration and extraction temperature; (**c**) effect of time and extraction temperature.

Response surface plots showing the effect of independent variables on the total polyphenols content of the extracts from Welsh onion leaves are shown in Figure 3. Overall, it was observed that an increase in the variables of the UAE (extraction temperature, time, and ethanol concentration) led to a greater content of total phenolic compounds in the extracts. When the temperature was increased at fixed time or ethanol concentration, the total polyphenols content of the extracts reached a maximum close to 60 °C (Figure 3b,c). Furthermore, it was observed that a longer extraction time was necessary at the lower temperature (30°C) to achieve a higher TPC content in the extract, while using the higher temperature (60 °C), a longer extraction time led to decrease the TPC content present in the extract of Welsh onion leaves.

Using the predictive polynomial for TPC (Equation (2)), it was possible to identify the following optimal conditions for the UAE of polyphenols from Welsh onion leaves: ethanol concentration: 70 % *v/v*, extraction time: 10 min, and temperature at 60 °C. These conditions allowed us to obtain extracts of Welsh onion leaves with total polyphenols content of 51.78 mg GAE/100 g. Tomšik et al. [43] obtained ethanolic *Allium ursinum* extracts with similar polyphenols content using the following optimal conditions for UAE: 80 °C temperature, 70% ethanol, 79.8 min, and 20.06 W/L ultrasonic power. González-de-Peredo et al. [44] identified the following optimal UAE conditions to maximize the extraction of both total phenolic compounds and total anthocyanins from red onion: 50% methanol as solvent, pH 2.0, 50 °C temperature, 30% amplitude, 0.4 s cycle, and 0.2:14 g sample/mL solvent ratio.

### 3.2. Effect of the UAE Conditions on the DPPH Radical Scavenging Activity

**Figure 4:**
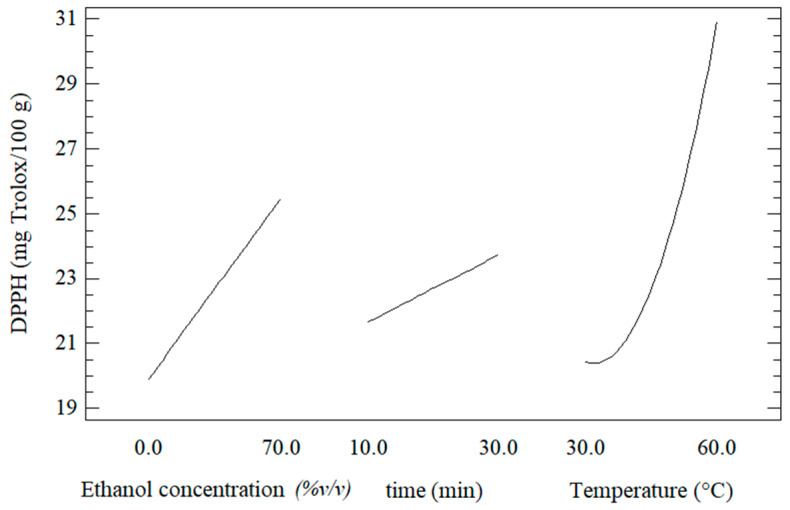
Effect of the individual variables process on the DPPH radical scavenging activity of the extracts of Welsh onion leaves.

Figure 4 shows the individual effect of the UAE conditions on the DPPH radical scavenging activity of the extracts of Welsh onion leaves. In general, it was observed that the DPPH radical scavenging activity of the extracts was increased at higher values of ethanol concentration, time extraction, and temperature. This behavior agrees with the increment of the concentration of phenolic compounds in the extracts (Figure 3).

Equation (3) shows the fitted second-order polynomial model for the DPPH radical scavenging activity of the extracts of Welsh onion leaves obtained from the response surface methodology.

(3) $$DPPH\;scavenging\;activity=-20.224+0.080x_{1}+0.534x_{2}-0.648x_{3}-0.010x_{2}x_{3}+0.013x_{3}{}^{2}$$

The results showed that the fitted model for the DPPH radical scavenging activity of the extracts of Welsh onion leaves had adjusted R^2^ around 83.30%. On the other hand, ANOVA indicated that the main factors (*x*_1_, *x*_2_, and *x*_3_) and the interactions between time-temperature (*x*_2_*x*_3_) and temperature-temperature (*x*_3_^2^) were the most significant parameters for obtaining an extract with a higher DPPH radical scavenging activity (*p* < 0.05) (Table 3). This behavior coincides with the same factors and most significant interactions for the case of TPC optimization (Table 2).

**Figure 5:**
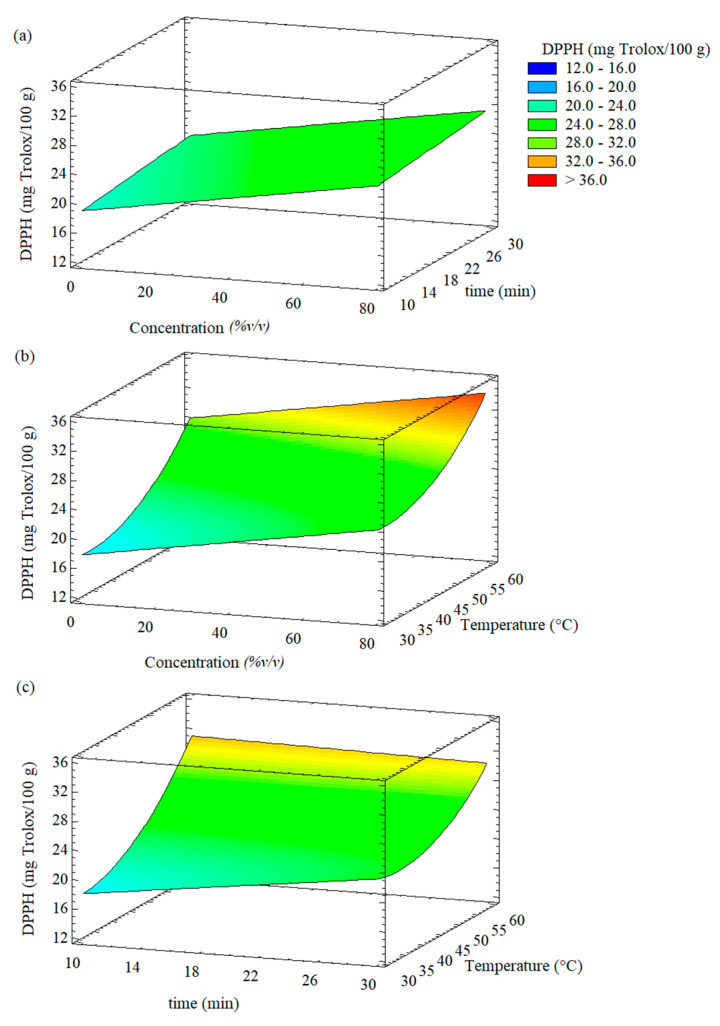
Response surface plots showing the effect of different experimental factors on the DPPH radical scavenging activity of the extracts from Welsh onion leaves: (**a**) effect of ethanol concentration and time; (**b**) effect of ethanol concentration and extraction temperature; (**c**) effect of time and extraction temperature.

Response surface plots showing the effect of independent variables on the DPPH scavenging activity of the extracts of Welsh onion leaves are shown in Figure 5. It was observed that the increase in the ethanol concentration and the extraction time led to greater values of DPPH scavenging activity (Figure 5a).

The effect of the ethanol concentration and temperature on the DPPH scavenging activity of the extracts of Welsh onion leaves is shown in Figure 5b. At a constant ethanol concentration, it was observed that with the increase in the extraction temperature, the DPPH scavenging activity increased until reached the highest value, and then decreased. This behavior was probably due to that the higher temperature improved the diffusion of ethanol through the onion matrix allowing better extraction [43,44]. Similar behavior was observed in the relationship between the DPPH scavenging activity and the time and temperature extraction (Figure 5c).

The optimal UAE conditions that maximized the DPPH scavenging activity of the extracts from Welsh onion leaves were 70% *v/v* ethanol, 60 °C, and 10 min. This allowed for the obtaining of extracts of Welsh onion leaves with DPPH scavenging activity of 34.07 mg Trolox/100 g. Bordin Viera et al. [45] obtained ethanolic *Allium cepa* L. extracts with DPPH scavenging activity of 23.52 mg Trolox/100 g using the following optimal UAE conditions: 25 °C, 20 min, 80% ethanol as solvent, and 750 W/L ultrasonic power.

### 3.3. Simultaneous Multi-Response Optimization

**Figure 6:**
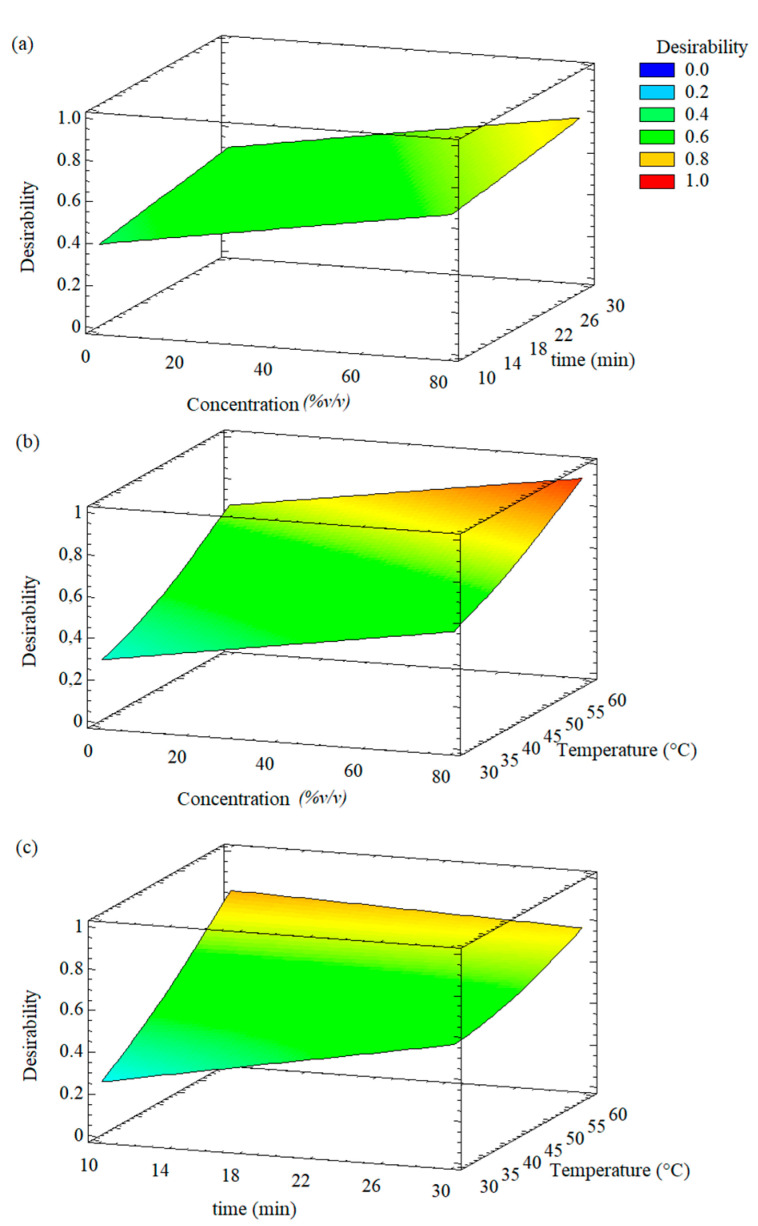
Response surface plots for simultaneous optimization of the total polyphenols content and DPPH radical scavenging activity of the extracts from Welsh onion leaves. (**a**) ethanol concentration and time; (**b**) ethanol concentration and extraction temperature; (**c**) time and extraction temperature.

The identification of the optimal UAE conditions for total polyphenols content and DPPH radical scavenging activity simultaneously was carried out by the desirability function (Figure 6). In this case, the only response variable was the desirability, with values between 0 and 1; the value of 1 represents the optimal configuration with which the greatest possible extraction of polyphenols and the DPPH scavenging activity would be achieved simultaneously, that is, it would be the desired value. It was observed that the desirability values increased as the variables of the process increased (Figure 6). In this sense, the best conditions of UAE extraction to reach the higher desirability were the same as the above-mentioned for the optimization of the total polyphenols content and the DPPH scavenging activity.

An experimental assay at the optimal conditions was carried out to validate the response variables predicted by the model. Table 4 shows the predicted and experimental results obtained at the optimal conditions. The error percentages between predicted and experimental results for the total polyphenols content and the DPPH radical scavenging activity were 0.9% and 5.0%, respectively. These results suggest that using RSM was a helpful technique for optimizing ultrasound-assisted extraction of polyphenols from Welsh onion leaves.

The extracts from Welsh onion leaves obtained at the optimal UAE conditions were selected for the following experiments involving antioxidant assays, UHPLC-ESI+-Orbitrap-MS analysis and FTIR spectroscopy.

### 3.4. Antioxidant Activity by ABTS and FRAP

The extracts from Welsh onion leaves obtained at the optimal UAE conditions were also evaluated by ABTS and FRAP methods to have a realistic assessment of their antioxidant activity [46,47]. The antioxidant activity determined through the ABTS assay was 155.51 ± 2.80 μM Trolox/100 g. Similar values were reported by Siddiq et al. to *Allium cepa* (120-133 μM Trolox/100 g) [48]. Regarding the antioxidant capacity determined by FRAP assay, the values observed were highest (1300.21 ± 65.55 μM Trolox/100 g) than those reported previously (819 μM Trolox/100 g) to calçot extracts [49].

### 3.5. Phenolic Composition for the Extracts at the Optimal UAE Conditions Obtained by UHPLC-ESI+-Orbitrap-MS Analysis

The phenolic compounds found in this work were similar to those reported in previous work for ethanolic extracts of Welsh onion leaves [3,8,14]. In general, cyanidin was the phenolic compound found in the highest amount followed by quercetin-3-glucoside (also known as isoquercetrin) (Table 5). This last flavonoid glycoside has been also previously identified and quantified by HPLC in ethanolic extracts from *Allium fistulosum* [14], and *Allium cepa* [50]. Tissut et al. [51] reported on the occurrence of quercetin 3-glucoside, kaempferol 3-glucoside, kaempferol 4′-glucoside, and isorhamnetin 4′-glucoside, as minor pigments from *Allium* cepa bulbs.

The most abundant phenolic acids in the extracts from Welsh onion leaves were ferulic acid and p-coumaric acid (Table 5). Tigu et al. [14] reported the lowest contents of ferulic acid in extracts of Welsh onion leaves (0.210 mg/kg). From the group of flavonols, quercetin was found in the highest amount followed by kaempferol (Table 5). These compounds have been detected in lower amount in extracts of Welsh onion leaves [14]. The other phenolic compounds analyzed were found below the quantification limit.

### 3.6. Chemical Conformation of the Extract Obtained at the Optimal UAE Conditions

**Figure 7:**
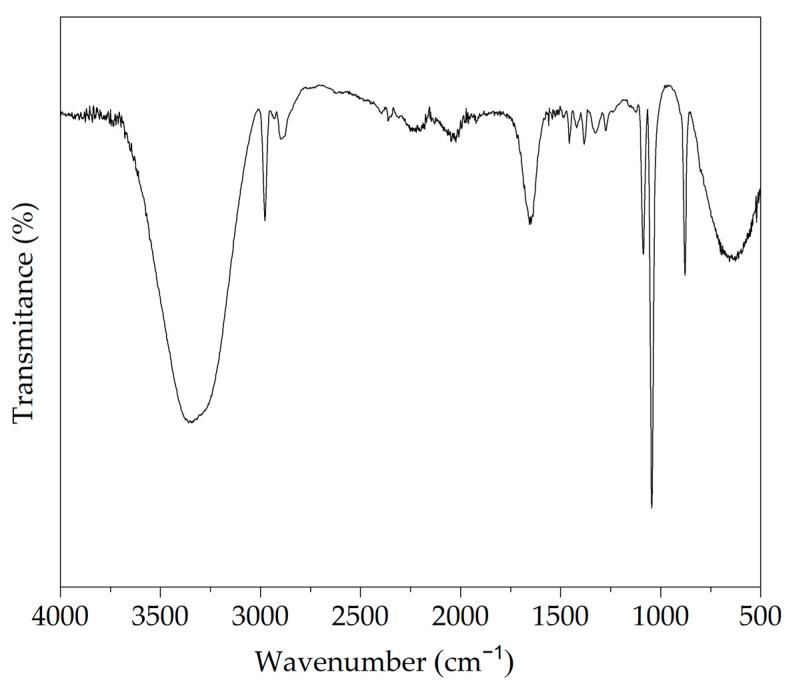
FTIR spectrum of the extract from Welsh onion leaves obtained at the optimal UAE conditions: extraction temperature of 60 °C, time of 10 min, and ethanol concentration of 70% *v/v*.

The identification of the main functional groups present in the extracts obtained under optimal UAE extraction conditions was carried out by FTIR (Figure 7).

In general, the infrared spectrum of the extracts obtained at the optimal UAE conditions showed typical functional groups of polyphenols-rich extracts [25]. The peaks located between 1450 cm^−1^ and 1300 cm^−1^ would indicate vibrations by stretching of the carbonyl groups [25]. The bands between 1500 cm^−1^ and 1400 cm^−1^ were attributed to the vibrations of the C–C bonds of phenolic groups [52]. The signal at 1650 cm^−1^ could be due to symmetrical and asymmetric vibrations of the carboxylate group (COO–) due to the presence of carboxylic groups [25]. The signals located at 1044 cm^−1^ and 1088 cm^−1^ were attributed to the presence of alcohol functional groups [28,53]. The peaks in the 3000-3300 cm^−1^ zone were attributed to the vibrations of the C–H bonds of the aromatic rings [53,54], for the bands between 1100 cm^−1^ and 1600 cm^−1^ correspond to the C–O and O–H vibrations of the polyphenols.

## 4. Conclusions

The ultrasound-assisted extraction conditions of polyphenols from Welsh onion leaves were successfully optimized using the response surface methodology. Ultrasonic treatment proved to be an efficient technique to generate changes in the microstructure of Welsh onion leaves causing the formation of microcracks and cell fragmentation. The extracts with highest values of total polyphenols content (51.78 mg GAE/100 g) and DPPH scavenging activity (34.07 mg Trolox/100 g) were obtained using an extraction temperature of 60 °C, time of 10 min, and ethanol concentration of 70% *v/v*. Cyanidin and quercetin-3-glucoside were identified as major components of the extracts obtained at the optimal conditions. The extracts from Welsh onion leaves obtained by UAE could be potentially used as natural antioxidants in food applications.

## Figures and Tables

**Table 1:** Complete factorial design (3^3^) employed to obtain the extracts from Welsh onion leaves by ultrasound-assisted extraction and experimental results.

n	Ethanol Concentration (% *v/v*) $x_{1}$	Extraction Time (min) $x_{2}$	Temperature (°C) $x_{3}$	TPC ^a,^* (mg GAE/100 g)	DPPH ^b,^* (mg Trolox/100 g)
1	35.0	10.0	30.0	16.87 (±1.66)	13.09 (±1.38)
2	0.0	10.0	30.0	25.32 (±1.56)	16.69 (±0.69)
3	70.0	30.0	30.0	40.35 (±1.97)	20.54 (±0.46)
4	0.0	30.0	30.0	41.14 (±0.43)	18.00 (±0.32)
5	0.0	20.0	30.0	38.95 (±0.58)	19.09 (±1.65)
6	35.0	20.0	60.0	45.05 (±2.68)	20.56 (±3.21)
7	0.0	10.0	45.0	41.03 (±0.47)	26.49 (±3.60)
8	35.0	30.0	60.0	42.32 (±1.29)	28.61 (±1.13)
9	0.0	30.0	60.0	40.43 (±2.07)	29.90 (±0.81)
10	70.0	20.0	45.0	20.56 (±4.12)	16.13 (±1.99)
11	70.0	30.0	45.0	27.85 (±1.75)	22.34 (±0.82)
12	70.0	30.0	60.0	42.88 (±4.28)	22.21 (±2.77)
13	35.0	20.0	45.0	47.47 (±4.17)	25.42 (±1.71)
14	35.0	10.0	60.0	44.06 (±2.79)	24.19 (±1.62)
15	0.0	20.0	45.0	46.29 (±1.71)	25.61 (±1.62)
16	70.0	20.0	60.0	46.52 (±4.21)	32.59 (±0.94)
17	0.0	10.0	60.0	44.89 (±1.15)	30.81 (±2.50)
18	70.0	20.0	30.0	45.76 (±0.54)	30.93 (±0.55)
19	70.0	10.0	30.0	36.30 (±1.10)	22.26 (±1.25)
20	35.0	20.0	30.0	40.05 (±0.59)	24.63 (±2.77)
21	70.0	10.0	45.0	46.33 (±1.46)	25.93 (±0.74)
22	0.0	20.0	60.0	39.93 (±2.44)	23.52 (±4.10)
23	35.0	30.0	45.0	43.53 (±0.95)	25.33 (±0.44)
24	35.0	10.0	45.0	43.15 (±2.40)	22.57 (±2.26)
25	0.0	30.0	45.0	50.99 (±1.59)	33.78 (±1.80)
26	35.0	30.0	30.0	53.83 (±2.36)	33.00 (±0.60)
27	70.0	10.0	60.0	50.08 (±2.11)	32.09 (±2.73)

^a^ Total polyphenols content; ^b^ DPPH radical scavenging activity; * Mean values ± standard deviation (n = 3).

**Table 2:** Analysis of variance (ANOVA) for total phenolic compounds.

Source	Sum of Squares	Degrees Freedom	Mean Square	Error	F-Value	*p*-Value
*x*_1_: Concentration	882.013	1	882.013	0.92113	77.00	0.0000
*x*_2_: time	590.346	1	590.346	0.92113	51.54	0.0000
*x*_3_: temperature	2374.35	1	2374.35	0.92113	207.28	0.0000
*x*_2_ *x*_3_	844.309	1	844.309	1.12815	73.71	0.0000
*x*_3_^2^	247.87	1	247.870	1.59545	21.64	0.0014
Lack of fit	1070.07	21	50.956		4.45	0.0000
Pure error	618.548	54	11.455			
Total	6627.51	80				

R^2^-(adjusted) = 72.82%, Standard error of estimation = 3.38446.

**Table 3:** Analysis of variance (ANOVA) for DPPH radical scavenging activity of the extracts of Welsh onion leaves.

Source	Sum of Squares	Degrees Freedom	Mean Square	Error	F-Value	*p*-Value
*x*_1_: Concentration	418.964	1	418.964	0.52780	111.40	0.0000
*x*_2_: time	60.3885	1	60.3885	0.52780	16.06	0.0002
*x*_3_: temperature	1484.14	1	1484.14	0.52780	394.64	0.0000
*x*_2_ *x*_3_	73.4735	1	73.4735	0.64642	19.54	0.0000
*x*_3_^2^	158.717	1	158.717	0.91418	42.20	0.0026
Lack of fit	204.384	21	9.73256		2.59	
Pure error	203.081	54	3.76077			
Total	2603.14	80				

R^2^-(adjusted) = 83.30%, Standard error of estimation = 1.93927.

**Table 4:** Experimental and predicted values of total polyphenols content and DPPH radical scavenging activity at the optimal UAE conditions.

Optimal Conditions			Results	
^a^ [Et] (% *v/v*)	^b^ time (min)	^c^ T (°C)	^d^ TPC (mg GAE/100 g)	^e^ DPPH (mg Trolox/100 g)
**Experimental values**				
70	10	60	51.33 (±2.54) *	32.35 (±1.47) *
**Predicted values**				
70	10	60	51.78	34.07

^a^ Ethanol concentration, ^b^ Extraction time, ^c^ Temperature, ^d^ Total polyphenols content and ^e^ DPPH radical scavenging activity. * Mean values ± standard deviation (n = 3) and wet basis.

**Table 5:** Main phenolic compounds identified in the extracts obtained at the optimal UAE conditions by UHPLC-ESI+-Orbitrap-MS analysis.

Phenolic Compounds	t_R_ (min)	Concentration (mg kg^−1^)
p-Coumaric acid	3.6	0.70 (±0.01)
Ferulic acid	3.7	1.85 (±0.21)
Quercetin	4.5	0.55 (±0.21)
Cyanidin	3.8	6.00 (±0.56)
Kaempferol	4.9	0.30 (±0.14)
Quercetin-3-glucoside	3.6	4.40 (±0.28)
Kaempferol-3-glucoside	3.5	1.20 (±0.14)

## Data Availability

The data presented in this study are available on request from the corresponding author.

## Supplementary Materials

The following supporting information can be downloaded at: https://www.mdpi.com/article/10.3390/foods11162425/s1, Table S1: Validation parameters of the assays of total polyphenols content and antioxidant activity, Figure S1: Chromatograms of main phenolic compounds identified in the extracts obtained at the optimal UAE conditions by UHPLC-ESI+-Orbitrap-MS analysis.
