# Supplementary material for: Improvement of the Ultrasound-Assisted Extraction of Polyphenols from Welsh Onion (Allium fistulosum) Leaves Using Response Surface Methodology
Source: Foods. 2022 Aug 12;11(16):2425. doi: 10.3390/foods11162425 (PMC9407235; doi:10.3390/foods11162425)
Supplement: Supplementary file 1 [file foods-11-02425-s001.zip › foods-1848687-supplementary.pdf]

foods

MDPI

Table S1. Validation parameters of the assays of total polyphenols content and antioxidant activity.

Validation parameters	TPC	DPPH	ABTS	FRAP
Regression equation	$y = 0.0111x + 0.0375$	$y = 0.57041x - 3.7571$	$y = 0.0428x + 9.7444$	$y = 0.001x + 0.0526$
Correlation coefficient (R^2)	0.9956	0.9882	0.9789	0.9679
Concentration range	4 - 90 mg/L	10 - 170 mg/L	100 - 2000 µM	100 - 500 µM
RSD*	4.176	4.21	1.80	5.04

* RDS: Relative Standard Deviation Intra-day.

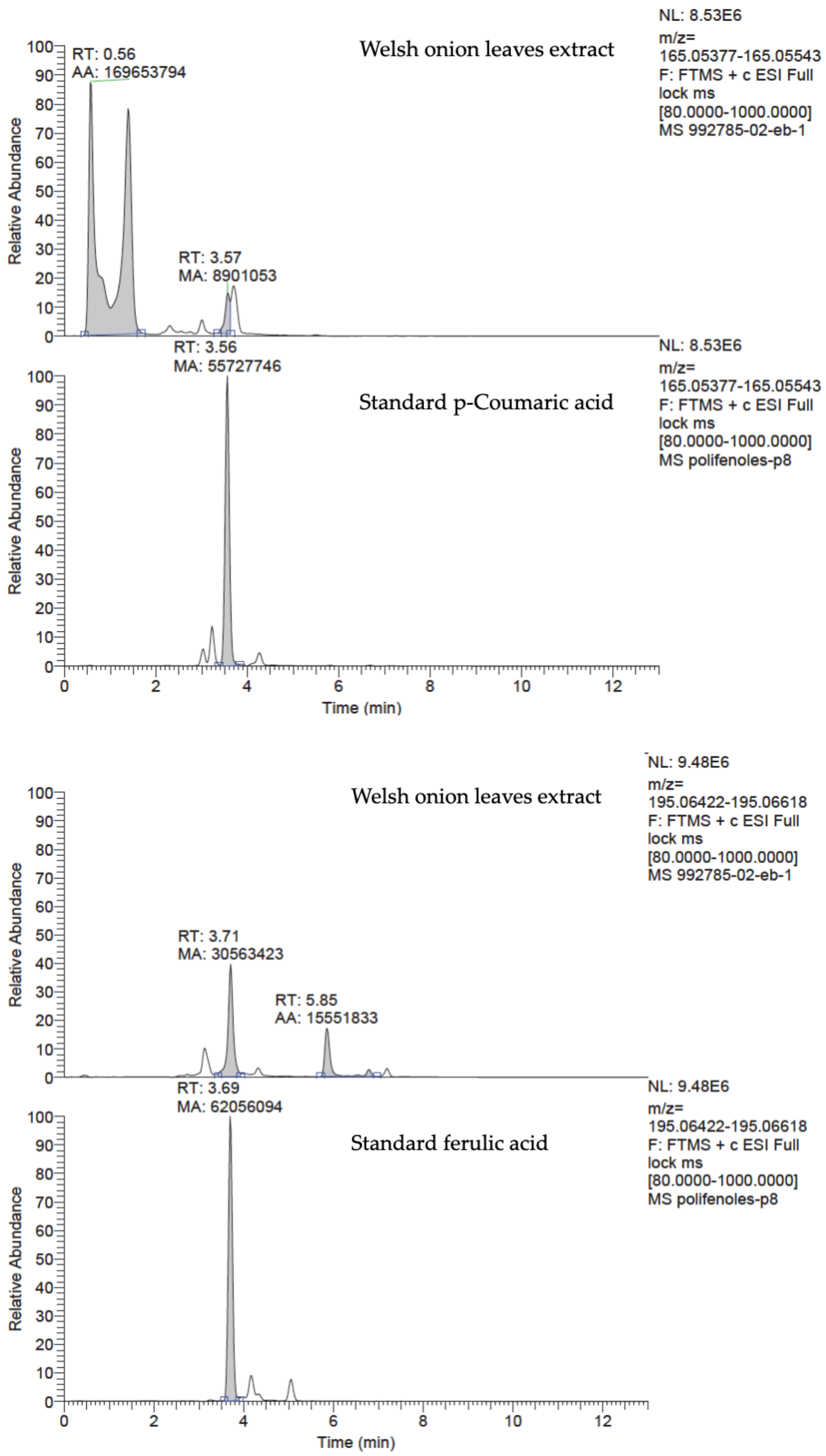

Figure S1. *Cont.*

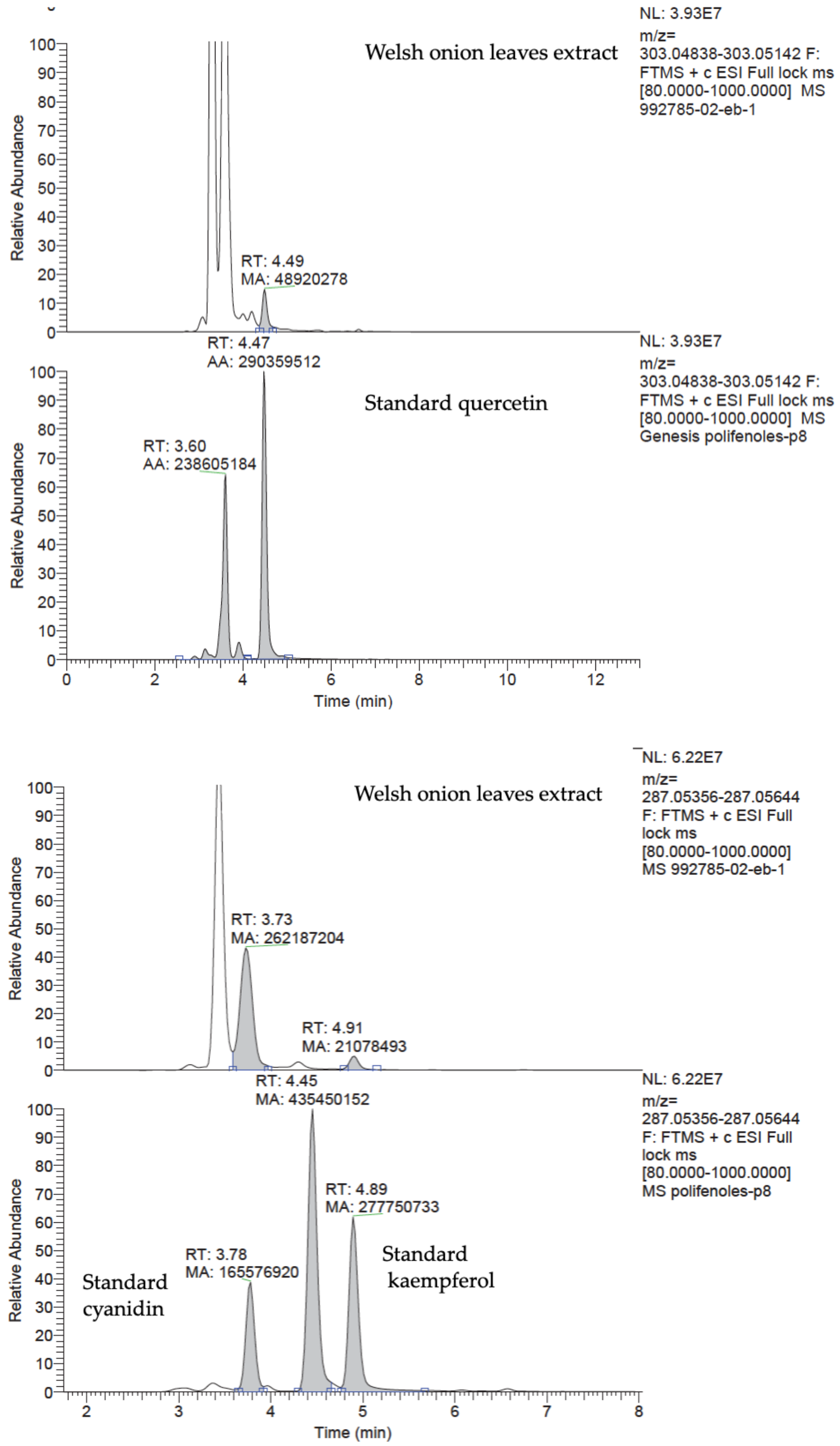

Figure S1. *Cont.*

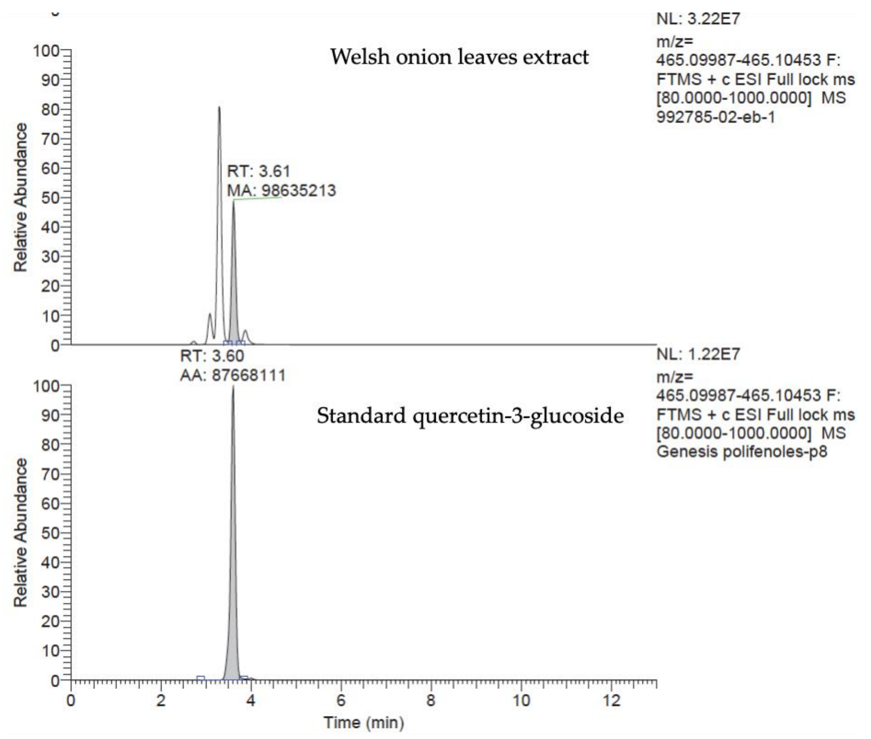

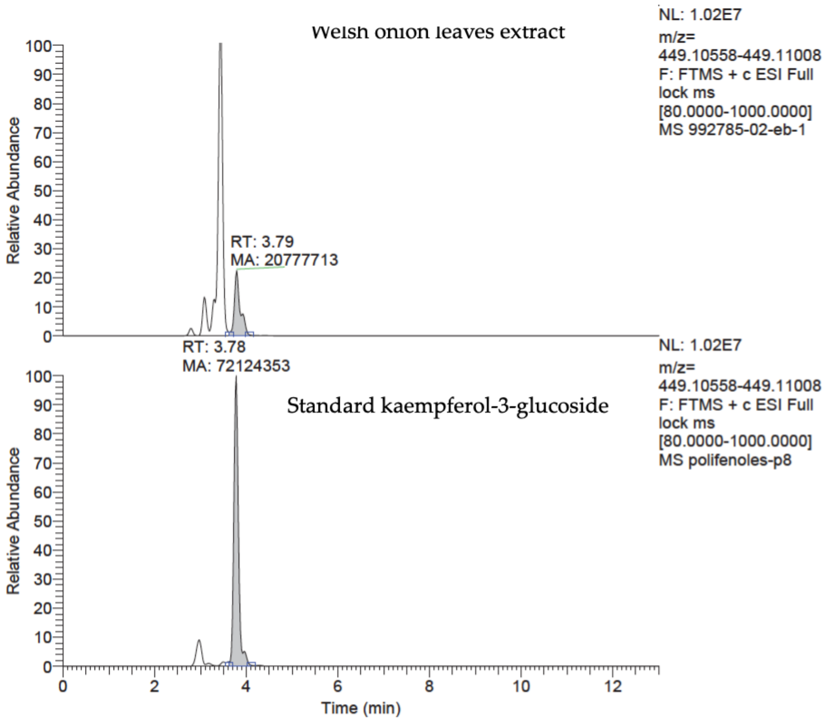

Figure S1. Chromatograms of main phenolic compounds identified in the extracts obtained at the optimal UAE conditions by UHPLC-ESI+-Orbitrap-MS analysis.
